# Supplementary material for: Dispositional cognitive effort investment and behavioral demand avoidance: Are they related?
Source: PLoS One. 2020 Oct 14;15(10):e0239817. doi: 10.1371/journal.pone.0239817 (PMC7556472; doi:10.1371/journal.pone.0239817)
Supplement: S1 Appendix — (DOCX) [file pone.0239817.s001.docx]

Dispositional Cognitive Effort Investment and Behavioral Demand Avoidance:
Are They Related?

Supplementary Material

Alexander Strobel^1^, Gesine Wieder^1^, Philipp C. Paulus^1,2^, Florian Ott^1^, Sebastian Pannasch^1^, Stefan J. Kiebel^1^ & Corinna Kührt^1^

^1^ Faculty of Psychology, Technische Universität Dresden

^2^ Max Planck Institute for Cognitive and Brain Sciences Leipzig

# Supplementary Methods

## Participants

Out of a total of 282 volunteers who had been screened by phone for the inclusion and exclusion criteria (see main text), eleven individuals had to be excluded based on inclusion/exclusion criteria. Further 47 individuals could not be invited for assessment due to conflicting schedules. The remaining 224 participants completed a personality questionnaire battery and worked on behavioral paradigms at two time points. Seven of these participants were excluded from further analyses due to failed data recordings ($n=5$) or non-compliance to the instruction ($n=2$) during the behavioral paradigms. Thus, the final sample comprised $N$ = 217 participants.

With regard to the relation of the present sample to that used in Kührt et al. (2019), please note the following: We used a sample of participants who were to come to our lab twice to complete personality questionnaires and performed two demand selection tasks as well as a cognitive task battery at both time points, with an interval between the assessments of about five weeks. At the first assessment, *N* = 244 participants took part, but only *N* = 217 took part in the second assessment. In Kührt et al. (2019), we used all participants who had taken part in the first assessment and only the personality data from this first assessment to replicate the integrative factor model of Cognitive Effort Investment established in a preceding study using Confirmatory Factor Analysis (CFA) of data from an unrelated larger sample assessed online. In the present study, we used a subsample of participants who had taken part in both assessments and used the personality data to relate them to the behavioral data from the experimental tasks, controlling for cognitive functioning measures derive from the cognitive task battery. The method used for this was latent state-trait modeling (see below). Thus, both with regard to the data used and the methods applied, we present new data. Only for the purpose of deriving personality factor scores for latent state-trait modeling, we referred to the CFA method, using less participants, but more data, i.e., from both time points, in one CFA model. Accordingly, in the present study, both the CFA models fitted and the results reported for factor loadings (see Figure S2) and model fit (see the Results section of the main manuscript) differ from that reported in Kürth et al. (2019), and are also not of primary interest here, but provided for reasons of completeness only.

## Demand Selection Task vs. COG-ED paradigm

We originally intended to use both the Demand Selection Task (DST) and the COG-ED paradigm as two measures of the same construct. In preparation and construction of the study we faced several points in favor of rather implementing two versions of the DST:

(1) To have two measures of the same construct was important for our aims, as latent state-trait (LST) modeling requires to have (at least) two measures of one construct at each time point for estimating the state-, method- and trait-specific variances in measures of the construct. This implies that the measures of the construct in question should not be too different. Given that the DST and the COG-ED have a quite different structure (demand avoidance vs. effort discounting), we reasoned that we would risk to have too low overlap of both tasks (i.e., behavior in these tasks may be not as highly correlated as required for LST modeling).

(2) We actually piloted both tasks in a number of participants, however, in a between design, which is why we do not have own data on a direct comparison between the DST and the COG-ED. Yet, valid conclusions about the existence of a correlation of demand avoidance in the DST with effort discounting in the COG-ED of, say, *r* ≥ .30 would have required a within-design with at least 80 participants, a piloting effort that was simply not manageable.

(3) We therefore had to decide whether we could construct two similar versions of the DST or two similar versions of the COG-ED and found the former more promising and manageable than the latter.

(4) In addition, during piloting, participants had expressed great discomfort with the COG-ED. Since we were dependent on the participants coming to the laboratory twice and did not drop out because of disliking the task, it seemed more sensible to us to use two variants of the DST.

## Cognitive task battery

To assess basic cognitive abilities such as fluid intelligence, processing speed, working memory, and switching ability that may have an impact on the choice behavior in the demand selection task, we employed a short cognitive task battery at the beginning of each appointment. The battery comprised the following tasks:

The *Digit-Symbol-Substitution Test* taken from the German version of the WAIS-III (von Aster, Neubauer, & Horn, 2006) was used to assess the speed of information processing that might have an influence on reaction times in the demand selection task. The participant is asked to substitute the digits 1 to 9 as fast as possible with symbols according to a substitution scheme printed on top of the page. Seven rows of 20 digits each are to be processed, with the total processing time of two minutes starting from the eighth digit. The outcome measure is the number of correctly substituted symbols and can take values of up to 133.

The *Digit Span Backwards Test*, also taken from the German version of the WAIS-III, was employed as a measure of working memory capacity. This test consists of seven tasks with two trials each that comprise an increasing number of digits (2-8). The digits are read to the participant, who is then to repeat them in reverse order. If the participant reports the correct result in at least one of the two trials, the next task is performed. Otherwise, the task stops and the outcome measure is the number of the last correctly performed task (1-7).

Two versions of the *Trail-Making Test* (see, e.g., Lezak, Howieson, Loring, Hannay, & Fischer, 2004) were used to examine mental speed (versions A and B) and task shifting ability (version B only). In version A, the participant is asked to connect 25 numbers scattered across a sheet of paper in ascending order. In version B the task is to connect numbers and letters in alternating order (i.e., 1-A-2-B …). The outcome measure is the time for completion of each version.

In addition, we assessed chrystallized intelligence by means of the *Multiple-Choice Vocabulary Test* (Lehrl, 2005). This test consists of 37 lists composed of one real German word and four pseudowords. The participants’ task was to identify the real word. The outcome measure is the number of correct answers.

Please note that we finally only used the Trail-Making Test A and B for our analyses (see below Statistical Analyses).

## Statistical analyses

R packages used. We used *RStudio* (Version 1.1.463; RStudio Team, 2016) with R (Version 3.6.1; R Core Team, 2018) and the R-packages *car* (Version 3.0.3; Fox & Weisberg, 2011), *lavaan* (Version 0.6.5; Rosseel, 2012), *psych* (Version 1.8.12; Revelle, 2018), *pwr* (Version 1.2.2; Champely, 2018), *robustbase* (Version 0.93.5; Todorov & Filzmoser, 2009), and *semTools* (Version 0.5.2; semTools Contributors, 2016) as well as *lsttheory* (Version 0.1-2; Mayer, 2014) for our analyses. This manuscript was created using RMarkdown with the packages *papaja* (Version 0.1.0.9842; Aust & Barth, 2018), *knitr* (Version 1.24; Xie, 2015), and *shape* (Version 1.4.4; Soetaert, 2018).

Demand avoidance: Original and new measure. The original demand avoidance measure was calculated as percentage of low-demand choices across all blocks (see Figure S1 for exemplary choice patterns of eight randomly selected participants). Following the suggestions made by Juvina et al. (2018), we also computed a new demand avoidance measure: We first determined the block-wise demand detection point (DDP) by employing a sliding window of size $n=12$ trials across individual decisions for patterns associated with high vs. low demand trial by trial starting with trial twelve. Using Wilcoxon sign tests, it was determined whether the individual choice rate in the respective window was significantly different from 0.5. If the Wilcoxon test was significant for a given trial and remained significant for all further trials, the DDP was set to the respective trial number. If the test yielded non-significant results for any further trial, the search for a DDP after that trial was started, and if a DDP could not be found throughout a given block, it was set to the highest trial number, i.e., 72. Choice after detection (CAD) was then determined as the percentage of low demand choices in the window from the DDP to the end of each block. In cases where the DDP was equal to the highest trial number, CAD was set to the total percentage of low demand choices across each block. The new demand avoidance measure was then determined as DDP – CAD, with DDP being the trial number where demand detection occurred and CAD being the rate of high-demand choices in the trials that followed the DDP. Both DDP and CAD were normalized by dividing the DDP by the total trial number per block, and by subtracting the rate of high-demand choices from one, respectively. The resulting new demand avoidance measure therefore ranged from -1 to 1.

Cognitive task battery. The variables originally considered were the number of correctly substituted symbols in the Digit-Symbol-Substitution Test (DSST), the digit span from the Digit Span Backwards Test (DSB), the time needed for completion of the Trail Making Test version A and B, respectively (TMTA and TMTB), and the number of correctly identified words in the Multiple-Choice Vocabulary Test (MWT) at both time points. Our plan was to estimate a two-factor model of cognitive functioning from these variables in order to have two more or less parallel measures of cognitive functioning for latent state-trait modeling. The reason for aiming at two measures was that we wanted to have symmetry to the personality an behavioral variables. However, due to the correlational pattern, this turned out to be not feasible. While the TMTA and TMTB scores correlated as highly as expected with each other at both measurement occasions, $r_{s}\geq$ .51, the correlations between the DSST, DSB and MWT scores were rather low, .01 $\leq r_{s}\leq$ .17, and the DSST and the DSB scores correlated only with the TMTA and TMTB scores to a sufficient degree, .14 $\leq|r_{s}|\leq$ .48. Given this constellation, we decided to use the TMTA and TMTB scores as the only indicators of cognitive functioning. Given that these measures capture individual differences in mental speed and task shifting as well as to some extent also working memory capacity (because being able to remember the location of numbers and/or letters yet to process will speed up subsequent trail-making), we considered these measures as sufficiently general measures of cognitive functioning.

# Supplementary Results

Figure S1 exemplifies individual choice behavior in the Demand Selection Task. Figure S2 gives the fitted confirmatory factor model of Cognitive Effort Investment. Figure S3 shows the correlations of the original and the new demand avoidance measure at both time points. Please refer to the main text for details.


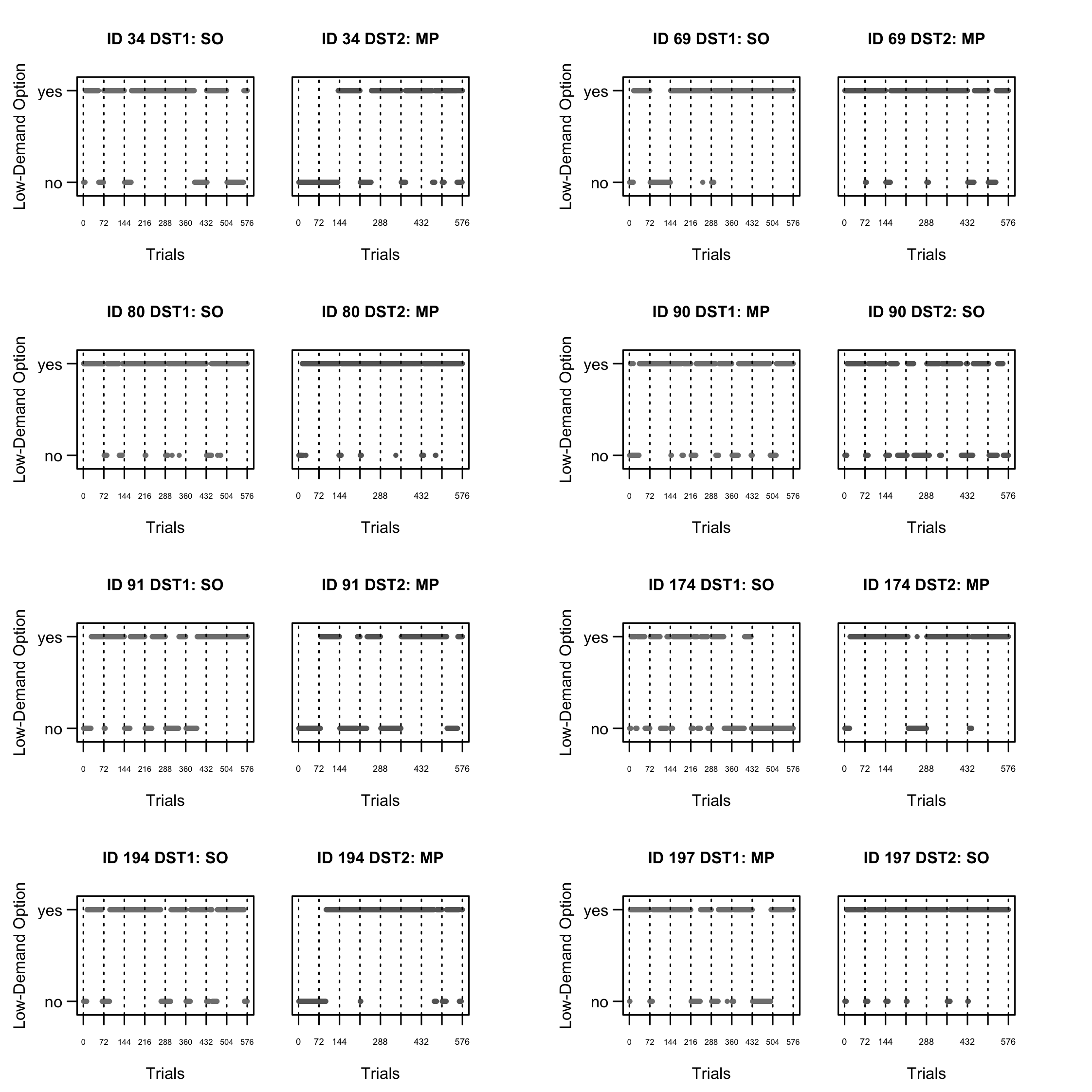


*Figure S1.* Demand Selection Task: Exemplary choice patterns from eight participants.


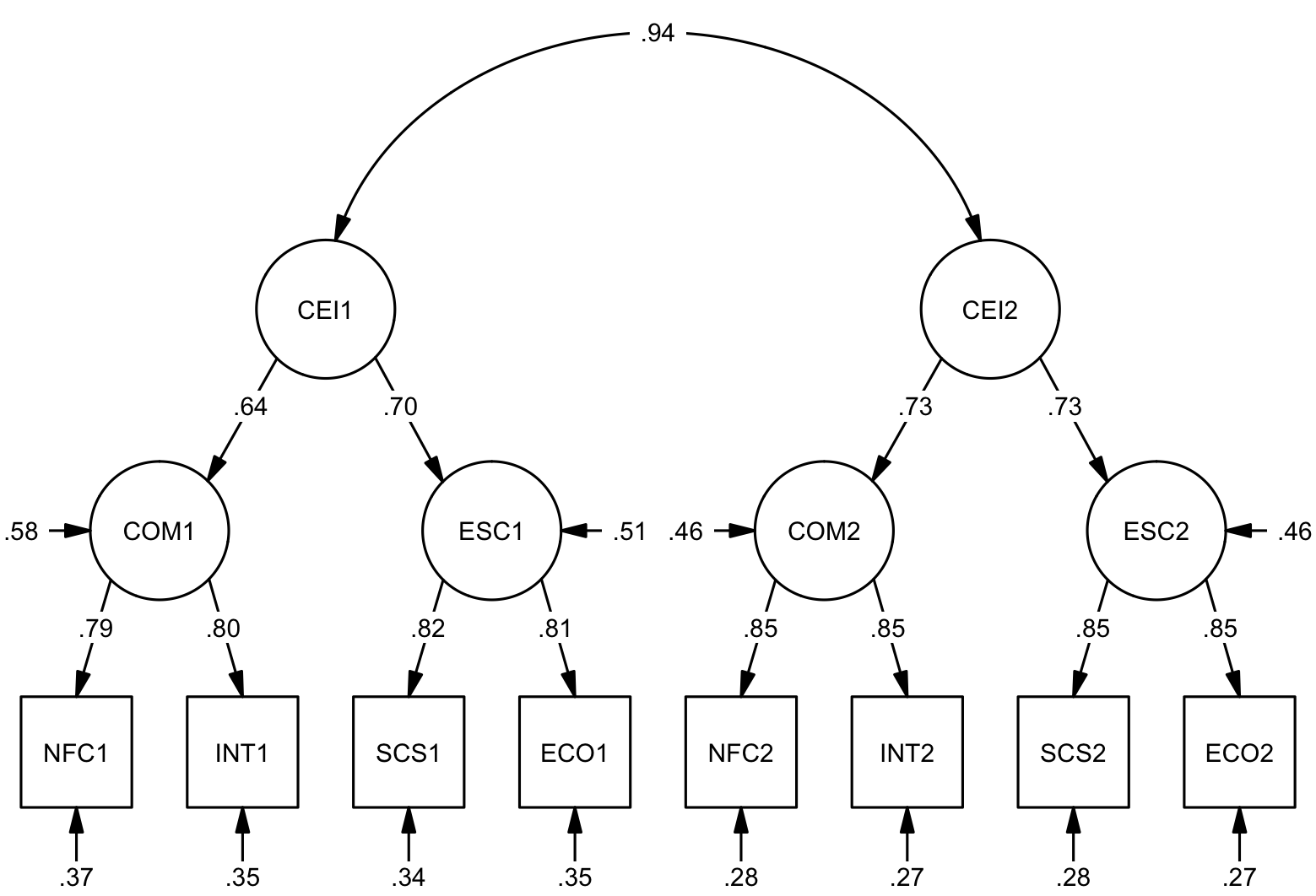


*Figure S2.* Hierarchical factor model of Cognitive Effort Investment (CEI) explaining the shared variance in Cognitive Motivation (COM), estimated from NFC = Need for Cognition and INT = Trait Intellect, and Effortful Self-Control (ESC), estimated from SCS = Self-Control and ECO = Effortful Control at the two time points 1 and 2.


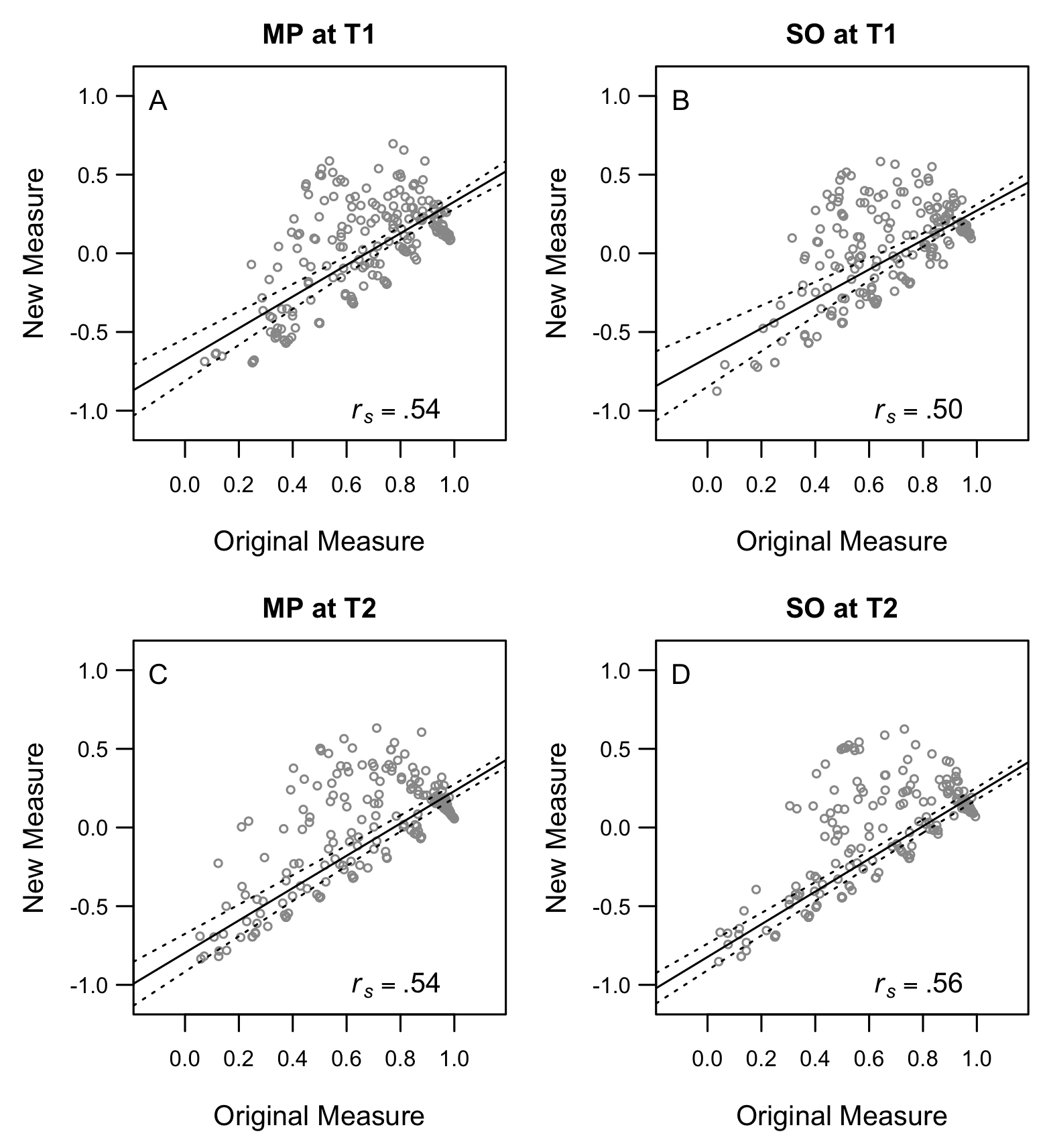


*Figure S3.* Spearman correlations of the original and the new demand avoidance measures. Lines give the robust fit together with 95% confidence intervals.

# References

Aust, F., & Barth, M. (2018). *papaja: Create APA manuscripts with R Markdown*. Retrieved from <https://github.com/crsh/papaja>

Champely, S. (2018). *Pwr: Basic functions for power analysis*. Retrieved from <https://CRAN.R-project.org/package=pwr>

Fox, J., & Weisberg, S. (2011). *An R companion to applied regression* (Second). Thousand Oaks CA: Sage. Retrieved from <http://socserv.socsci.mcmaster.ca/jfox/Books/Companion>

Kührt, C., Pannasch, S., Kiebel, S. J., & Strobel, A. (2019). Dispositional individual differences in effort investment: Etablishing the core construct. *PsyArXiv*. Available from: <https://psyarxiv.com/rwz62/>.

Juvina, I., Nador, J., Larue, O., Green, R., Harel, A., & Minnery, B. (2018). Measuring individual differences in cognitive effort avoidance. In T. Rogers, M. Rau, X. Zhu, & C. W. Kalish (Eds.) (pp. 1886–1891). Austin, TX: Cognitive Science Society.

Lehrl, S. (2005). *Mehrfachwahl-Wortschatz-Intelligenztest MWT-B*. Balingen: Spitta Verlag.

Lezak, M. D., Howieson, D. B., Loring, D. W., Hannay, H. J., & Fischer, J. S. (2004). *Neuropsychological assessment*. New York: Oxford University Press.

Mayer, A. (2014). *lsttheory: Latent state-trait theory*. Retrieved from <https://github.com/amayer2010/lsttheory>

R Core Team. (2018). *R: A language and environment for statistical computing*. Vienna, Austria: R Foundation for Statistical Computing. Retrieved from <https://www.R-project.org/>

Revelle, W. (2018). *Psych: Procedures for psychological, psychometric, and personality research*. Evanston, Illinois: Northwestern University. Retrieved from <https://CRAN.R-project.org/package=psych>

Rosseel, Y. (2012). lavaan: An R package for structural equation modeling. *Journal of Statistical Software*, *48*(2), 1–36. Retrieved from <http://www.jstatsoft.org/v48/i02/>

RStudio Team. (2016). *RStudio: Integrated development environment for R*. Boston, MA: RStudio, Inc. Retrieved from <http://www.rstudio.com/>

semTools Contributors. (2016). *semTools: Useful tools for structural equation modeling*. Retrieved from <https://CRAN.R-project.org/package=semTools>

Soetaert, K. (2018). *Shape: Functions for plotting graphical shapes, colors*. Retrieved from <https://CRAN.R-project.org/package=shape>

Todorov, V., & Filzmoser, P. (2009). An object-oriented framework for robust multivariate analysis. *Journal of Statistical Software*, *32*(3), 1–47. Retrieved from <http://www.jstatsoft.org/v32/i03/>

von Aster, M., Neubauer, A., & Horn, R. (2006). *Wechsler Intelligenztest für Erwachsene WIE. Deutschsprachige Bearbeitung und Adaptation des WAIS-III von David Wechsler*. Frankfurt: Pearson Assessment.

Westbrook, A., Kester, D., & Braver, T. S. (2013). What is the subjective cost of cognitive effort? Load, trait, and aging effects revealed by economic preference. *PLoS ONE, 8*(7), e68210.

Xie, Y. (2015). *Dynamic documents with R and knitr* (2nd ed.). Boca Raton, Florida: Chapman; Hall/CRC. Retrieved from <https://yihui.name/knitr/>
