# Supplementary material for: Dispositional cognitive effort investment and behavioral demand avoidance: Are they related?
Source: PLoS One. 2020 Oct 14;15(10):e0239817. doi: 10.1371/journal.pone.0239817 (PMC7556472; doi:10.1371/journal.pone.0239817)
Supplement: Attachment — Submitted filename: Response to Reviewers.docx [file pone.0239817.s002.docx]

PONE-D-19-33696

Dispositional cognitive effort investment and behavioral demand avoidance: Are they related?

**Response to Reviewers**

In the following, we provide authors’ responses (AR) for all points raised by the editorial office (EP) and two reviewers (RP). We consider the questions raised by the former serious and believe that we could convincingly answer them. We also greatly appreciate the comments of the latter and hope that we were able to adequately address all the issues raised. Changes to the manuscript/supplemental material are highlighted by red font.

**Editorial office**

**EP:** Did the authors present any new data in this submission that were not previously presented in the published article Kührt et al. (2019)? Did the authors perform any additional experiments or collect any additional data that were not a part of the study from the published article Kührt et al. (2019)?

**AR:** A clear yes to both your questions. We present new data, did perform additional experiments and collected additional data that were not part of the manuscript by Kührt et al. (2019), which is presently only openly available as a preprint on *PsyArXiv* (<https://psyarxiv.com/rwz62/>), but is under review at another journal. We apologize for obviously not having acknowledged clearly enough the relation of our submission to the manuscript by Kührt et al. (2019). In our submission, we wrote (p. 8, lines 172-176): “Please note that this sample is a subsample of the sample used in Study 2 in Kührt et al. [22] where we replicated the hierarchical factor model of Cognitive Effort Investment. The present sample consists of those participants who took part in both assessments and had complete data for all measures relevant for the present report. There is no duplicate reporting of results.”

In more detail: Both manuscripts are part of a larger project on individual differences in effort investment and adjustments in cognitive control. Study 1 in Kührt et al. (2019) was the first study of this project, our manuscript is about the second study of this project. In Kührt et al. (2019), we aimed at establishing a factor model of Cognitive Effort Investment that integrated the traits Need for Cognition and Self-Control in one overarching construct. This laid groundwork for the second study described in the present submission, where we aimed at relating behavioral demand avoidance to one overarching construct, i.e., Cognitive Effort Investment, instead of two or even more, thereby potentially increasing type I error rates. Thus, although there is a common theme across both manuscript, they differ with regard to data sets, research questions and analyses. Merging both manuscripts would have resulted in a very long and potentially inaccessible paper about two different research questions.

With regard to the sample used in our study, we had a sample of participants who came to our lab twice to complete personality questionnaires and performed two demand selection tasks as well as a cognitive task battery at both time points, with an interval between the assessments of about five weeks. At the first assessment, *N* = 244 participants took part, but only *N* = 217 took part in the second assessment. In Kührt et al. (2019), we used all participants who had taken part in the first assessment and only the personality data from this first assessment to replicate the integrative factor model of Cognitive Effort Investment established in a preceding study using Confirmatory Factor Analysis (CFA) of data from an unrelated larger sample assessed online. In the present study, we used a subsample of participants who had taken part in both assessments and used the personality data to relate them to the behavioral data from the experimental tasks, controlling for cognitive functioning measures derive from the cognitive task battery. The method used for this was latent state-trait modeling. Thus, both with regard to the data used and the methods applied, we present new data. Only for the purpose of deriving personality factor scores for latent state-trait modeling, we referred to the CFA method, using less participants, but more data, i.e., from both time points, in one CFA model. Accordingly, in the present submission, both the CFA models fitted and the results reported for factor loadings (S1 Appendix: Figure S2) and model fit (p. 19, lines 408-409) differ from that reported in Kürth et al. (2019), and are also not of primary interest, but provided for reasons of completeness only.

We hope we could make clear that while having one common theme—dispositional cognitive effort investment—both studies target different research questions, primarily use different methods and that our submission presents new data, analyses and results not published previously. We have included the content of the previous paragraph in the Supplementary Material under Supplementary Methods (and refer to this in the main manuscript, p. 8, line 176) to be as transparent as possible also to all readers interested in our study.

**Reviewer #1**

**RP1.1:** Admittedly, I am not a personality researcher and the statistical methods employed here (e.g., CFA) would be better evaluated by someone from this research community. However, as someone with a strong interest in cognitive effort, I found myself wondering why this study was done— what particular gap in the effort literature does this work address? Perhaps this is a matter of the authors providing a better motivation for their efforts, but the best I could find was: “to determine the trait-like nature of both self-reported Effort investment and behavioral Demand avoidance in order to systematically relate stable individual differences in these measures to each other” (p 7). Why is this important to do? For example, for cognitive effort researchers, what exactly is staked on the validity of these self-report measures? Would any researcher whole relies primarily upon behavioral or task-based measures—rather than self-report—be surprised at the lack of relationship ? Overall, I found the introduction to be lengthy but I found that they key motivation for this study (and accordingly, its possible contribution within the literature) gets a bit ‘lost in the shuffle.’

**AR1.1:** We agree with the reviewer that from the original introduction it can be difficult to understand the purpose of the study. We apologize for not having motivated clearly the point of our study. We aimed at validating an often-made assertion in the literature. This assertion is that self-reported self-control (and similar traits related to dispositional effort investment) are related to behavioral demand avoidance. In our view, this is important because it is all too often assumed that measuring a personality characteristic at one point in time informs about the trait level of that characteristic and, critically, that correlates of that characteristic will also be trait-like. For example, in their highly influential paper Westbrook et al. (2013) state that “lesser effort discounting predicted greater self-reported engagement with cognitively demanding activities using the NCS. This correlation supports: 1) a trait-like property of effort costliness […] and 2) that our measure captures that trait.” (Westbrook et al., 2013, p. 7). In the field of interindividual differences research, this is quite a critical statement with potentially far-reaching consequences: If this were the case, then the behavioral measure may qualify as a time-stable predictor for real-life outcomes. However, it could also be the case that the behavioral measure just happened to be correlated with the personality measure for some state influences at the point in time both were measured, which would compromise the predictive nature of the behavioral measure.

Although Westbrook et al. (2013) and Kool et al. (2013) explicitly and implicitly argue that the correlations observed (between Need for Cognition and effort discounting as in Westbrook et al., 2013, or between Self-Control and demand avoidance as in Kool et al., 2013) point to some trait-like behavioral characteristic in the measures obtained via the COG-ED paradigm or the demand selection task (DST), this trait-likeness has not been explicitly shown yet. To establish whether performance in these tasks is trait-like, we determined the trait-like nature of at least one measure of demand avoidance/effort discounting (see AR1.2) and related the stable trait-variance in these measures to the stable trait variance in self-reported cognitive effort investment. Our aims were thus two-fold: First, to establish that behavioral demand avoidance was trait-like at all, and second, to test whether trait variance covaries with the trait variance in self-reported cognitive effort investment.

We believe our findings to be valuable for a wider community of cognitive effort researchers. To derive predictions about real-life outcomes from some experimental measures, it would be highly relevant to relate the experimental measures to self-report data of typical effort-related behavior in situations that require the exertion of mental effort. To do so validly, we believe that it is also of interest to have some proxies for the stable, trait-like nature of both the behavioral measures and the self-report data that may be related to the former. Our results show that measures of behavioral demand avoidance display enough stable trait variance to be of importance as predictors of real-life outcomes, but that – at least in our experiments – there is no evidence for trait-covariation between behavioral and self-report measures.

Of course, one may question whether self-report and behavioral data need to show some correspondence. We firmly believe that they should, because otherwise one could not draw reliable conclusions on behavioral patterns originating from assumed stable individual differences. In summary, we agree with the reviewer that we did not made clear enough the intention and therefore the novelty of our study and now properly motivate our study in the introduction (pp. 6-7, lines 114-143).

**RP1.2:** Oddly one of the key task-based measures noted in the introduction (p 1) is the COG-ED. Yet The authors report that they refrained from using the COG-ED measure (a somewhat well-established measure of effort avoidance vis-à-vis discounting) because of “the rather low correspondence to be expected of this measure and the DST and instead employed two versions of the DST” (p 6). This analysis doesn’t appear to be reported anywhere in this submission (I assume the authors did it in some prior version of this study?), but getting COG-ED choice data seems important given the importance placed on this measure at numerous points in the manuscript. Isn’t it documented by Westbrook et al. (2013) that NFC correlates well with COG-ED-assessed discounting rates.

**AR1.2:** We thank the reviewer for bringing up this key point. Indeed, we originally intended to use both the Demand Selection Task (DST) and the COG-ED paradigm as two measures of the same construct. In preparation and construction of the study we faced several points in favor of implementing only the DST:

(1) To have two measures of the same construct was important for our aims, as latent state-trait (LST) modeling requires to have (at least) two measures of one construct at each time point for estimating the state-, method- and trait-specific variances in measures of the construct. This implies that the measures of the construct in question should not be too different. Given that the DST and the COG-ED have a quite different structure (demand avoidance vs. effort discounting), we reasoned that we would risk to have too low an overlap of both tasks (i.e., behavior in these tasks may be not as highly correlated as required for LST modeling).

(2) We actually piloted both tasks in a number of participants, however, in a between design, which is why we do not have own data on a direct comparison between the DST and the COG-ED. Yet, valid conclusions about the existence of a correlation of demand avoidance in the DST with effort discounting in the COG-ED of, say, *r* ≥ .30 would have required a within-design with at least 80 participants, a piloting effort that was simply not manageable.

(3) We therefore had to decide whether we could construct two similar versions of the DST or the COG-ED and found the DST task more promising and manageable than the COG-ED task.

(4) In addition, during piloting, participants had expressed great discomfort with the COG-ED. Since we were dependent on the participants coming to the laboratory twice and did not drop out because of disliking the task, it seemed more sensible to us to use two variants of the DST.

Interestingly, our decision was reinforced when we learned during informal talks at conferences that apparently, the DTS and the COG-ED are not substantially correlated to each other. Indeed, in hindsight, we might have arrived at other conclusions had we used two variants of the COG-ED. Still, as stated in the Discussion section of the manuscript, one has to consider that the correlation between effort discounting and Need for Cognition observed by Westbrook et al. (2013) remains to be replicated, and so far, we found only one replication attempt in a children sample. In our opinion, our theoretical and methodological consideration of the Westbrook et al. (2013) study and their COG-ED paradigm in the current version of the main manuscript addresses the points outlined above, but still holds a balance between informing the readers and avoiding too detailed information on a task not used. For those interested in this issue in more detail, we elaborate on these reasons, as outlined above, in the Supplemental Material (S1 Appendix: Supplementary Methods, pp. 2-3).

**RP1.3:** The authors contend this work is a conceptual replication of Kool et al. 2013. In what way is this a conceptual replication of this study? Their work presents an analysis of neuroimaging/behavioral data. The connection between this study and their is not made readily apparent to readers.

**AR1.3:** We apologize that we did not make this clear enough. A prominent part of the work of Kool et al. (2013) is about a synthesis of neuroimaging data on self-control and cognitive costs. In the empirical part, however, Kool et al. (2013) show demand avoidance to be related to self-reported self-control. It is this specific result that we aimed at replicating conceptually. Apart from also providing a *direct* replication (i.e., the bivariate correlation of self-control and the demand avoidance measure used by Kool et al., 2013, see p. 21 of our manuscript), we conceptually replicate the result by using a more general measure of dispositional cognitive effort investment and a more general measure of demand avoidance derived from two similar tasks. In order to make our approach clearer, we rephrased and clarified our research aim (p. 7, lines 140-143).

**Reviewer #2**

**RP2.1:** I find it surprising that the new measure of demand avoidance (i.e., the one that considers demand detection) does not seem to differ much from the old measure of demand avoidance. What was the procedure for determining the demand detection point?

**AR2.1:** We thank the reviewer for making this point as it allows us to clarify the procedure used. As detailed in the Supplemental Material (see S1 Appendix: Supplementary Methods, pp. 5-6), we closely followed the reviewer’s procedure: Following the suggestions made by Juvina et al. (2018), we first determined the block-wise demand detection point (DDP) by employing a sliding window of size 𝑛 = 12 trials across individual decisions for patterns associated with high vs. low demand trial by trial starting with trial twelve. Using Wilcoxon sign tests, it was determined whether the individual choice rate in the respective window was significantly different from 0.5. If the Wilcoxon test was significant for a given trial and remained significant for all further trials, the DDP was set to the respective trial number. If the test yielded non-significant results for any further trial, the search for a DDP after that trial was started, and if a DDP could not be found throughout a given block, it was set to the highest trial number, i.e., 72. Choice after detection (CAD) was then determined as the percentage of low demand choices in the window from the DDP to the end of each block. In cases where the DDP was equal to the highest trial number, CAD was set to the total percentage of low demand choices across each block. The new demand avoidance measure was then determined as DDP – CAD, with DDP being the trial number where demand detection occurred and CAD being the rate of high-demand choices in the trials that followed the DDP. Both DDP and CAD were normalized by dividing the DDP by the total trial number per block, and by subtracting the rate of high-demand choices from one, respectively. The resulting new demand avoidance measure therefore ranged from -1 to 1.

This new demand avoidance measure was correlated at r_s_ ≥ .50 with the original measure (see S1 Appendix: Supplementary Results, p.9 Figure S3). In order to make sure that the resulting measure was not different when using a different sliding window, we also performed new analyses with other sizes of the sliding window, *n* = 9 and *n* = 18. This did not essentially impact on the results (i.e., the correlations between changed only at the second or third digit after the decimal and did not reach significance in any case). This information is now given as footnote to the procedure for determining the demand detection point (see S1 Appendix: Supplementary Methods, p. 5).

**RP2.2:** In the section “Personality-behavior relationships are weak at best”, last paragraph, the authors mention “the task was designed to demonstrate—and actually instructed to induce—the avoidance of cognitive demand”. Please clarify what you meant by “actually instructed to induce”, because as far as I know most studies using DST (including Kool’s original ones) do not attempt to bias subjects toward demand avoidance.

**AR2.2:** We thank the reviewer for giving us the opportunity to elaborate on this issue. Indeed, in Experiment 1 of the paper by Kool et al. (2010), the instruction was as follows: “Subjects were told that they were free to choose from either deck on any trial and that they should ‘feel free to move from one deck to the other whenever you choose’ but also that ‘if one deck begins to seem preferable, feel free to choose that deck more often’.” (Kool et al., 2010, p. 667). In Kool et al. (2013), a similar instruction was given: “Participants were told they were free to sample from either target, but that if they developed a preference they should feel free select one more than the other.” (Kool et al., 2013, p. 4). During initial pilot experiments, we actually used a more neutral instruction and could not find a clear demand avoidance pattern. This is consistent with evidence from your own research (Juvina et al., 2018) where demand avoidance was less pronounced when using an instruction that did not direct participants’ attention towards a difference between the choice options or indirectly suggested to choose one option more often. In contrast, when adhering to the instruction of Kool et al. (2010) in further pilot experiments, we were able to find a bias towards low demand choices and therefore used it in the main experiment. We elaborate on this issue in the revised version of the manuscript (Methods section, p. 11, lines 231-235, and Discussion section, p. 30, lines 632-633).
