# Supplementary material for: Dispositional cognitive effort investment and behavioral demand avoidance: Are they related?
Source: PLoS One. 2020 Oct 14;15(10):e0239817. doi: 10.1371/journal.pone.0239817 (PMC7556472; doi:10.1371/journal.pone.0239817)
Supplement: Attachment — Submitted filename: Response to Reviewers.docx [file pone.0239817.s003.docx]

PONE-D-19-33696R2

Dispositional cognitive effort investment and behavioral demand avoidance: Are they related?

**Response to Reviewers**

In the following, we provide authors’ responses (AR) for the points raised by the reviewers (RP). We hope that we were able to adequately address all the issues raised. Changes in the manuscript are highlighted by red font.

**Reviewer #1**

**RP1.1:** I still believe more work needs to be done in the manuscript to address my previous comment (AR1.1)—the authors’ response does a good job clarifying the purpose of this study but the revised Introduction doesn’t really make this underlying motivation clear. To this end, I think the introduction would benefit from some restructuring. I was also confused by the mention of ‘real-life outcomes’ in the added text to page 7. What are these real-life outcomes and how can we measure them?—trait measures, task-based measures, or neither?

**AR1.1:** We thank the reviewer for the opportunity to further improve the structure of the Introduction and clarify thereby the underlying motivation. We agree that in the previous version of the Introduction, the actual motivation for the study becomes only apparent on page 6, which seems too late.

In the revised version, we state our research question already in the first paragraph of p. 4. We then continue with discussing the problem that the relation of two putative traits cannot be determined using only measures per trait at one point in time, but rather using latent state-trait modeling (the remainder of p. 4). However, to do so, one needs two parallel measures each per construct – here self-reported cognitive effort investment and behavioral cognitive effort avoidance (pp. 4-5). We then outline the parallel behavioral measures originally intended and afterwards consider the question of whether NFC and Self-Control are parallel self-report measures of cognitive effort investment (pp. 5-7).

We believe that in this revised form, the purpose of our study becomes more obvious early in the Introduction. We hope that readers with no background in personality and individual differences will find this structure an easier access to our research.

Regarding ‘real-life outcomes’ and how to measure them, we detailed this by rewriting the respective sentence (now p. 4, lines 66-69): “If so, then such behavioral measures would qualify as time-stable predictors for real-life outcomes such as self-control failures in everyday life as measured via ecological momentary assessment (e.g., [12]) or relapse from addiction treatment (e.g., [13]). The cited references give examples of studies that used behavioral measures as predictors of real-life outcomes: Wolff et al. (2016) found that behavioral measure of executive function predict real-life self-control failures, and Kräplin et al. (2020) observed that behavioral measures impulsive decision-making predicts the course of addictive disorders.

**Reviewer #3**

**RP3.1:** The results reported here are, admittedly, a bit disappointing for me, but they are not surprising. I spoke with the lead author at a conference a while ago, and we talked about this work. I pointed him towards the recent work by Juvina, who computed an alternative measure of demand avoidance in the DST, but unfortunately this seems to not have lead to any qualitative changes in the results. Regardless of my personal feelings, I think the authors have performed a valiant effort and I applaud them for their experimental and statistical rigor.

**AR3.1:** We thank again for this comment and thank the reviewer very much for his overall assessment of our work.

**RP3.2:** The only difference I can see between our implementation of the DST and the version that this team uses (aside from the consonant/vowel - capital/non-capital manipulation), is that we deliberately never tell our participants how well they are performing. As we detailed in the 2010 paper that introduced this task, we never do this because we want to avoid that participants are using an error-avoidance rather than an effort-avoidance strategy. In fact, the 2010 paper describes some pain-staking efforts to rule out that participants’ behavior on this task is driven by a desire to reduce error commission. Therefore, I was surprised to see that this team did not only include accuracy feedback at the end of each block, but also that they provided encouragement either affirming that their current behavior was desirable or pushing for a change. It is therefore possible that in the current version of the DST the choice behavior can’t be seen as a pure measure of pure effort avoidance, but that it reflects error avoidance (or a desire to perform well) instead. It is important to note that effort and error avoidance are not necessarily related to each other. Therefore, I think that, at the very least, the section named “self-reported cognitive effort investment and behavioral demand avoidance are unrelated” should include a discussion of this possibility.

**AR3.2:** We thank the reviewer for this very insightful comment. Yes, it seems we did not consider a possible bias arising from performance feedback/encouragement. To address the possibility that choice behavior in our implementation of the DST was to some extent driven by an error avoidance strategy, we reanalyzed our data as follows: For each participant, DST version, and time point, we predicted the average choice behavior across blocks 2 to 8 by the average hit rate during the preceding block by means of a linear mixed model, allowing for random intercepts and slopes per individual. Hit rates during the previous block did not significantly predict choice behavior in the current block (all p > .182). We now report these results in the Results section (lines 443-449) and, as the reviewer suggests, we also discuss this issue in the Discussion section under “Self-reported cognitive effort investment and behavioral demand avoidance are unrelated” (lines 585-591). Even in the absence of a significant effect of previous performance on current choice behavior, it remains a limitation that we did not establish a task environment identical to that of the original DST.

**RP3.3:** Related to this point I want to mention that when we run the task, we always aim to not push people in any direction, only telling them that they can sample freely between the decks, but that they can choose one more if the prefer it. Note that this latter instruction doesn’t necessarily push them towards the least effort option, as the authors seem to insinuate. It is completely possible that participants take it as meaning they can choose cues based on their location or visual appearance. In contrast, the current version of this task does provide some signal that people could interpret as a push to change their behavior in a consistent way (through the accuracy feedback).

**AR3.3:** We thank the reviewer for pointing out this difference to the original task. Yet, we do think that the original instruction “if one deck begins to seem preferable, feel free to choose that deck more often” (Kool et al., 2010, p. 667) may be taken by some participants as some suggestion to behave in a certain way. This notion is fostered by our own pilot experiments and by the results of study 1 by Juvina et al. (2018): with a more neutral instruction, demand avoidance was less pronounced. Still, we agree with the reviewer that the original instruction does not necessarily *push* participants to choose the low effort option more often as one sentence in our Discussion section suggested. It originally read: “While interindividual variation exists, the distribution of low demand choices is shifted towards a higher propensity for demand avoidance, because the task was designed to demonstrate a general avoidance of cognitive demand and the original instruction even suggests that one should feel free to choose a preferable deck more often.” While the reviewer surely agrees to the first part of the sentence, the second (underlined) part of the sentence is a debatable interpretation. We therefore deleted the underlined part in our revision (p. 29, lines 639-641).

**RP3.4:** Finally, I found this paper rather hard to read. Upon some reflection, this is primarily caused by the excessive use of abbreviations, acronyms and initialisms (especially in the tables and figures). I simply do not have the working memory capacity needed to maintain them all (and I think most other readers do not have either). Would the paper be hurt by spelling out variables by their full name? To be honest, I think this would greatly improve readability. For example, the terms “reliability, trait consistency, etc.” are not named very often, so I am not sure they need to have an abbreviation. Moreover, if the authors end up deciding to include some of them, there should be more consistency. Sometimes variables for demand avoidance start with only a D (e.g., DMP2), and at other times with DA (e.g., DAo). Anything that can be done here would be greatly appreciated.

**AR3.4:** We apologize for our inflationary use of abbreviations and tried our best to remove any unnecessary abbreviations from the manuscript. In all tables, the variables are now spelled out, the abbreviations for reliability, trait consistency etc. are removed as well. Only in the figures, we kept abbreviations due to limitations in space.

**RP3.5:** Line 187 - The variable intellect seems to not measure intellect per se (“the faculty of reasoning and understanding objectively, especially with regard to abstract or academic matters”), but rather something akin to need for cognition. I think the term used for this measure should reflect this (e.g., “interest in intellect”, “motivation for intellect”).

**AR3.5:** We fully agree with the reviewer that the term “intellect” may not be the best label for the trait measure in question. Yet, this term was not coined by us, but was used by Mussel (2013) in his effort to provide a framework that structures traits related to intellectual engagement and curiosity. The term “intellect” traces back to a distinction of two aspects of one of the traits of the five factor model of personality (e.g., McCrae & Costa, 1997), Openness to Experience. DeYoung et al. (2007) proposed that each of the five factors of personality is composed of two aspects. For Openness to Experience, they proposed that these aspects are Openness and Intellect. Openness in the narrow sense refers to the aspect of the broader concept of Openness to Experience that captures individual differences related to open-mindedness with regard to aesthetics, imagination and fantasy. Intellect on the other hand refers to ingenuity and openness to ideas or values, and this term has been used in some of the progenitors of the currently accepted five factor model of personality. We are afraid that elaborating on this issue would go beyond the scope of our manuscript, and we do not see us in the position to rename this variable for our purposes.

**RP3.6:** Is the variable pertaining to cognitive functioning made up completely of the Trail Making Test? Wouldn’t be better to name this measure something closer to that variable? Given its broad name, it now seems to measure something more general.

**AR3.6:** We agree that the label “cognitive functioning” leaves the impression of a far more general measure of cognitive ability that we actually have when using only two versions of the Trail Making Test. Indeed, we originally aimed at having a broader measure of cognitive ability derived not only from the Trail Making Test, but also from other measures such as the Digit Span Backwards and the Digit-Symbol Substitution tests taken from the Wechsler Adult Intelligence Scale III. Yet, it turned out that the indices of cognitive ability derived from the latter tests did not correlate as strongly with each other and with the indices of the two Trail Making Test versions as expected. It was therefore not possible to use all measures to create two comparable measures of cognitive functioning that we needed for latent state-trait modeling (see the Supplementary Material for details). Actually, we had some debate on how to term this variable given that it eventually was only composed of the two Trail Making Test versions. Yet, the Trail Making Test version A measures mental speed and to some extent working memory, while the Trail Making Test version B in addition requires shifting, and hence, central cognitive correlates of general mental ability are captured by this task. Therefore, we finally decided to use the term “cognitive functioning” to refer to a variable that clearly captures central aspects of general mental ability, but does not evoke the idea of a broader concept like the original intended “cognitive ability”. We hope that it now becomes clearer why we chose this very term.

**RP3.7:** Line 336 - What is “demand avoidance cognitive functioning”?

**AR3.7:** Thank you for noticing this. This actually was a mistake, it should have read “demand avoidance and cognitive functioning”. We corrected it in the revised version of this manuscript.

**References**

DeYoung, C. G., Quilty, L. C., & Peterson, J. B. (2007). Between facets and domains: 10 aspects of the Big Five. *Journal of Personality and Social Psychology, 93*(5), 880-896.

Kool, W., McGuire, J. T., Rosen, Z. B., & Botvinick, M. M. (2010). Decision making and the avoidance of cognitive demand. *Journal of Experimental Psychology: General, 139*(4), 665-682.

Juvina, I., Nador, J., Larue, O., Green, R., Harel, A., & Minnery, B. (2018). Measuring individual differences in cognitive effort avoidance. In T. Rogers, M. Rau, X. Zhu, & C. W. Kalish (Eds.), *Proceedings of the 40th Annual Conference of the Cognitive Science Society* (pp. 1886-1891). Austin, TX: Cognitive Science Society.

McCrae, R. R., & Costa, P. T. (1997). Personality trait structure as a human universal. *American Psychologist, 52*(5), 509-516.

Mussel, P. (2013). Intellect: a theoretical framework for personality traits related to intellectual achievements. *Journal of Personality and Social Psychology, 104*(5), 885-906.
